# Prevalence of gastro-oesophageal reflux disease, and its associated risk factors among medical students: a nation-based cross-sectional study

**DOI:** 10.1186/s12876-023-02899-w

**Published:** 2023-08-07

**Authors:** Mohamed Baklola, Mohamed Terra, Amr Badr, Fayrouz Mohamed Fahmy, Enas Elshabrawy, Yousef Hawas, Doaa Abdel-Hady, Abdel-Hady El-Gilany

**Affiliations:** 1 Faculty of Medicine, Mansoura University, 60El-Gomhoria Street, Mansoura, 35516 Egypt; 2 Cardiology Department, Benha Teaching Hospital, Banha, Egypt; 3 Faculty of Medicine, Menofia University, Menofia, Egypt; 4 Faculty of Medicine, Tanta University, Gharbeya, Egypt; 5 Public Health and Community Medicine Department, Faculty of Medicine, Mansoura University, 60El- Gomhoria Street, Mansoura, 35516 Egypt

**Keywords:** Gastroesophageal reflux disease, Medical students, Prevalence, Risk factors

## Abstract

**Background:**

Gastroesophageal reflux disease (GERD) is a common digestive problem in adults particularly medical students, who are one of the most vulnerable groups. Many variables, including lifestyle changes and psychological stress, increase the prevalence of GERD among undergraduate medical students. Therefore, this study aims to assess the prevalence, and risk factors of GERD, and its relationship with perceived stress among medical students in Egypt.

**Methods:**

In November and December of 2022, a cross-sectional descriptive study with an analytical component was carried out among medical students from six different universities. An online self-reported questionnaire was used to collect data. The questionnaire included data on sociodemographic characteristics, risk factors, lifestyle, the Arabic version of GerdQ, and the Arabic version of Cohen’s Perceived Stress Scale (PSS).

**Results:**

The questionnaire was filled out by 964 medical students, the majority of whom were female (64%). Overall, 17.1% of participants reported symptoms of GERD. Logistic regression showed that smoking, high perceived stress, and a family history of GERD were the independent predictors of having GERD symptoms, with odds ratios of 4.1, 3.9, and 2.2, respectively.

**Conclusion:**

GERD is a frequent condition among Egyptian medical students, affecting around one-fifth of them. In the fight against GERD, university-based smoking cessation and stress management programs may be influential.

## Introduction

Gastroesophageal reflux disease (GERD) is a chronic disorder characterised by the regurgitation of stomach contents into the oesophagus. GERD is predicted to impact 13.98% of the adult population worldwide [1]. Patients with gastroesophageal reflux disease (GERD) typically present with regurgitation and heartburn following a meal; they also describe a burning sensation in the retrosternal area that may radiate to the neck or the back; heartburn may worsen when lying supine or bending over; and other, less common symptoms of GERD include dysphagia, chest pain, water brash, pain during swallowing, burping, hiccups, nausea, and vomiting [2].

In the absence of alarm symptoms, the diagnosis of GERD is primarily made based on symptoms. Patients with symptoms of GERD, such as heartburn and regurgitation, can be diagnosed and treated empirically with proton pump inhibitors (PPIs) [3].

The GerdQ questionnaire is a helpful tool, with a sensitivity of 65% and specificity of 71%, for guiding the diagnosis based on symptoms [4]. If symptoms persist despite PPI treatment or patients presented with extraoesophageal symptoms, further diagnostic investigations may be necessary. Endoscopy, pH monitoring, and esophageal manometry are reserved for such cases [3].

Causes of GERD include anatomical factors like hiatal hernia and functional factors such as poor lower oesophageal sphincter (LES) resting tone and acid clearance, as well as transient LES relaxation [5]. Obesity, dietary factors like coffee, spicy food, and carbonated beverages, smoking, non-steroidal anti-inflammatory drug (NSAID) use, alcohol intake, and psychological stress are all modifiable risk factors for the development of GERD [6].

The symptoms of GERD have been shown to be exacerbated by exposure to psychosocial stressors [7]. An increase in heartburn symptoms was found in a study of people who already suffer from heartburn when they were exposed to stress [8]. Although their actual acid levels are within the usual range, anxious people have a more exaggerated perception of acid events. It is theorised that stress makes patients more sensitive to acid reflux, which may explain why it seems to exacerbate symptoms [7]. The consequences of gastroesophageal reflux disease (GERD) can be extremely serious, including diminished quality of life and an increased risk of contracting other diseases [5].

Many factors put medical students at increased risk for gastroesophageal reflux disease (GERD), including an unhealthy diet, and excessive coffee intake [9]. A unique combination of psychological factors and lifestyle changes occurs when students enter undergraduate training in various disciplines. According to previous studies, medical undergraduate students, in particular, experience significant levels of stress and perceived stress [10, 11]. Medical students in the Middle East and Asia have a higher rate of anxiety than their peers worldwide, with 1 in 3 students suffering from anxiety [12]. A study conducted in Saudi Arabia reported that 25.9% of medical students suffer from GERD [13].

To the best of the authors’ knowledge, there are no studies on the prevalence and associated risk factors of GERD in the Medical student in Egypt, despite being one of the most vulnerable groups. So, the objectives of the current study were to estimate the prevalence of gastroesophageal reflux disease among medical undergraduates in Egypt and determine the risk factors.

## Materials & methods

### Study design and study period

A descriptive, cross-sectional study with an analytic component was conducted among Egyptian medical students from six universities. The data was collected from November to December 2022. This study was written in compliant with the STROCSS 2021 criteria [14].

### Sample size

Sample size was calculated using MedCalc 15.8 (https://www.medcalc.org/). The primary outcome of interest is the percentage of the students who had GERD. A previous study found that it was 14.8% in medical students [15], considering an alpha error of 5%, study power of 80%, and 5% precision with design effect 3. So, the minimum required sample size is 576 students.

### Sampling and data collection approach

A convenience sampling approach was taken after determining the required sample size. Using Google forms, respondents completed the questionnaire. The first-year students were excluded because they were not exposed yet to the factors related to medical school.

The questionnaire was delivered to all students through official groups via the Telegram app and other social media platforms. They could answer anonymously, on their own time, and were not compensated. Data was collected from six universities, to which the authors had access, named: Benha University, Mansoura University, Tanta University, Menofia University, Cairo University, and Kafr-elsheikh University.

### Study tools

The questionnaire is divided into five sections: social and demographic data, risk factors and lifestyle, the Arabic version of GerdQ for the diagnosis of GERD, as well as the Arabic version of Cohen’s Perceived Stress Scale (PSS). GerdQ is a reliable questionnaire for determining the likelihood of GERD. It has six questions: four positive questions to measure GERD symptoms (heartburn, regurgitation, sleep disturbance caused by heartburn and regurgitation, and medication use) and two negative ones (epigastric pain and nausea). Each item was scored from 0 to 3 based on the frequency of symptoms in the previous week. Those with a GerdQ score of > 8 were considered to have GERD. The Arabic version of GerdQ was developed and validated for use among Arabic speakers[16]. This questionnaire has a sensitivity of 65% and a specificity of 71% [17].

The Perceived Stress Scale (PSS) is a standard tool for assessing stress. Although the tool was created in 1983, it is still a popular choice for understanding how diverse situations affect feelings and perceived stress. This scale’s questions inquire about thoughts and emotions during the previous month. Grading PSS into low, moderate, and high was done as the following: scores ranging from 0 to 13 were considered low stress; scores ranging from 14 to 26 were considered moderate stress; scores ranging from 27 to 40 were considered high perceived stress [18].

### Statistical analysis

The Statistical Package for Social Science Program (SPSS 25 for Windows) was used to analyse the data. Descriptive statistical measures (i.e., percentage, frequency, mean, and standard deviation) were used to summarise the demographic characteristics of the participants, perceived stress, and other risk factors. The Pearson chi-square test was performed to investigate the relationship between different variables and the presence of GERD symptoms. Finally, univariate and multivariate binary logistic regression were used to model the association between various risk factors, and the presence of GERD symptoms. The adjusted odds ratio was used to calculate each variable’s risk to the specified factors. The result is considered statistically significant when the p-value is less than 0.05.

## Results

### Socio-demographic characteristics and prevalence of GERD

The questionnaire was filled out by 964 medical students with female participants making up the majority (64%) of those who took part. The prevalence of gastroesophageal reflux disease (GERD) was found to be 17.4% (n = 168) among study participants. The percentage of female participants with GERD was 66.7%, which was a little higher than the females without GERD symptoms. The participants’ mean age was 21.03 ± 1.7, and the academic year was estimated to be insignificant, with GERD frequency ranging from 22.6% in second-year students to 13.7% in sixth-year students. Moreover, a notable proportion of students (53%) showing GERD symptoms reported having a family history of GERD. This finding demonstrated a statistically significant difference when compared to those without any family history of GERD. The rest of the participants’ characteristics are detailed in Table 1.

**Table 1:** Sociodemographic characteristics of the participants and its association with GERD

		Total n (%)	No n (%)^*^	Yes n (%)^*^	P-value
Overall		**964**	**796 (82.6)**	**168 (17.4)**	
Sex	Female	617 (64)	505 (63.4)	112 (66.7)	0.43
	Male	347 (36)	291 (36.6)	56 (33.3)	
Age	Less than 20	219 (22.7)	187 (23.5)	32 (19)	0.17
	20–23	641 (66.5)	529 (66.5)	112 (66.7)	
	More than 23	104 (10.8)	80 (10.1)	24 (14.3)	
Academic Year	Second	254 (26.3)	216 (27.1)	38 (22.6)	0.36
	Third	206 (21.4)	166 (20.9)	40 (23.8)	
	Fourth	171 (17.7)	147 (18.5)	24 (14.3)	
	Fifth	219 (22.7)	176 (22.1)	43 (25.6)	
	Sixth	114 (11.8)	91 (11.4)	23 (13.7)	
Residence	Rural	452 (46.9)	365 (45.9)	87 (51.8)	0.16
	Urban	512 (53.1)	431 (54.1)	81 (48.2)	
Marital status	Single	953 (98.9)	787 (98.9)	166 (98.8)	0.94
	Married	11 (1.1)	9 (1.1)	2 (1.2)	
Family history of GERD	No	658 (68.3)	569 (71.5)	89 (53)	**< 0.01**
	*Yes*	306 (31.7)	227 (28.5)	79 (47)	

* Column Percentage, Yes = GerdQ score of > 8, No = = GerdQ score of < = 8

### Perceived stress and its relation to GERD

Table 2 shows that 736 (76.3%) of the 964 students who completed the survey had low to moderate perceived stress, whereas 228 (23.7%) had high perceived stress. Of those with GERD symptoms, 47% reported high perceived stress which is significantly higher than those without GERD symptoms (18.7%).

**Table 2:** Risk factors and lifestyle and their association with GERD

		Total n (%)	No n (%)^*^	Yes n (%)^*^	P-value
Overall		**964**		**168 (17.4)**	
*Perceived Stress*	*Low to moderate*	736 (76.3)	647 (81.3)	89 (53)	**< 0.01**
	*High*	228 (23.7)	149 (18.7)	79 (47)	
*BMI*	*Underweight/ Healthy Weight*	584 (60.6)	476 (59.8)	108 (64.3)	0.28
	*Overweight /Obese*	380 (39.4)	320 (40.2)	60 (35.7)	
*Physical activity (> 30 min) / week*	*No*	437 (45.3)	355 (44.6)	82 (48.8)	0.31
	*Yes*	527 (54.7)	441 (55.4)	86 (51.2)	
*Using Analgesics*	*No*	202 (21)	173 (21.7)	29 (17.3)	0.19
	*Yes*	762 (79)	623 (78.3)	139 (82.7)	
*Number of daily meals*	*Less than 3*	470 (48.8)	376 (47.2)	94 (56)	**0.04**
	*3 or more*	494 (51.2)	420 (52.8)	74 (44)	
*Preferred drink*	*Tea*	291 (30.2)	239 (30)	52 (31)	0.84
	*Coffee*	242 (25.1)	196 (24.6)	46 (27.4)	
	*Soda*	153 (15.9)	128 (16.1)	25 (14.9)	
	*Others*	12 (1.2)	11 (1.4)	1 (0.6)	
	*Don’t drink any of these*	266 (27.6)	222 (27.9)	44 (26.2)	
*Number of Coffee Cups/ day*	*Less than 3*	894 (92.7)	746 (93.7)	148 (88.1)	**0.014**
	*3 or more*	70 (7.3)	50 (6.3)	20 (11.9)	
*Eating dinner one or two hours before sleeping*	*No*	440 (45.6)	363 (45.6)	77 (45.8)	0.95
	*Yes*	524 (54.4)	433 (54.4)	91 (54.2)	
*Eating meals at midnight*	*No*	437 (45.3)	361(45.4)	76 (45.2)	0.97
	*Yes*	527 (54.7)	435 (54.6)	92 (54.8)	
*Quick eating habit*	*No*	490 (50.8)	406 (51)	84 (50)	0.81
	*Yes*	474 (49.2)	390 (49)	84 (50)	
*Frequency of breakfast/ Week*	*Less than 3*	361 (37.4)	287 (36.1)	74 (44)	0.14
	*3*	356 (36.9)	207 (26)	54 (23.9)	
	*More than 3*	247 (25.6)	302 (37.9)	40 (32.1)	
*Wearing tight clothes*	*No*	801 (83.1)	660 (82.9)	141 (83.9)	0.75
	*Yes*	163 (16.9)	136 (17.1)	27 (16.1)	
*Smoking*	*Non smoker*	925 (96)	774 (97.3)	151 (89.9)	**< 0.01**
	*Current smoker*	39 (4)	22 (2.7)	17 (10.1)	

*Column Percentage, Yes = GerdQ score of > 8, No = = GerdQ score of < = 8

**Table 3:** Binary logistic regression analysis of having GERD among study participants

			Positive on GerdQ*			
Variables		**Univariate Regression**			**Multivariate Regression**	
		**OR (C.I)**		**P-Value**	**AOR (C.I)**	**P-Value**
*Family history of GERD*	*No*	1 (r)		**< 0.01**	1 (r)	**< 0.01**
	*Yes*	2.2 (1.6–3.1)			2.2 (1.5–3.2)	
*Perceived Stress*	*Low to moderate*	1 (r)		**< 0.01**	1 (r)	**< 0.01**
	*High*	3.85 (2.7–5.5)			3.9 (2.7–5.6)	
*Number of daily meals*	*Less than 3*	1 (r)		**0.04**	1 (r)	0.21
	*3 or more*	0.7 (0.5–0.9)			0.8 (0.6–1.13)	
*Number of Coffee Cups/ day*	*Less than 3*	1 (r)		**0.012**	1 (r)	0.10
	*3 or more*	2.01 (1.2–3.5)			1.6 (0.9–3)	
*Smoking*	*Non smoker*	1 (r)		**< 0.01**	1 (r)	**< 0.01**
	*Current smoker*	3.9 (2.1–7.6)			4.1 (2–8.4)	

*GerdQ score of > 8

### GERD-related risk factors and lifestyle

Nearly half of the participants (45.3%) performed no physical activities for 30 min or more per week. Of those with GERDS symptoms, 48.8% of them never did physical activities.

There was no significant difference in the analgesics usage; 82.7% of those who experienced GERD symptoms used analgesics, whereas 78.3% of the participants who did not experience GERD symptoms reported using analgesics.

Regarding the number of daily meals, it was found to be a significant risk factor. Participants who reported eating three meals or more each day and suffering from GERD were 74 (44%) much lower than those who eat three meals or more and did not report GERD 420 (52.8%).

Coffee consumption was identified as a significant risk factor; 11.9% of participants who showed GERD symptoms drink three or more cups of coffee, compared to 6.3% of those who drink coffee three times or more per day and did not show GERD symptoms.

The prevalence of current smokers among the participants is notably low, with only 4%. Among participants experiencing symptoms of GERD, 10% were identified as current smokers, which is significantly higher compared to the prevalence of current smokers (2.7%) among those without GERD symptoms.

### Predictors of GERD

Univariate regression analysis was conducted to assess the associations between various variables and having GERD symptoms. Subsequently, multivariate regression analyses were performed to control for potential confounding factors and determine adjusted odds ratios (AORs).

Regarding the family history of GERD, participants without a family history served as the reference group. Participants with a positive family history of GERD showed a significantly increased likelihood of having GERD symptoms, with an odds ratio of 2.2. The association remained statistically significant after adjusting for other variables, yielding an AOR of 2.2.

In terms of perceived stress, participants with low to moderate stress levels were used as the reference group. Individuals experiencing high levels of perceived stress demonstrated a significantly higher probability with an odds ratio of 3.85. After adjustment for other variables, the association remained significant, resulting in an AOR of 3.9.

Regarding smoking status, non-smokers were used as the reference group. Current smokers exhibited a significantly increased probability of GERDQ positivity, with an odds ratio of 3.9. After adjustment for other variables, the association remained statistically significant, yielding an AOR of 4.1.

In the analysis considering the number of daily meals and the number of coffee cups consumed per day, participants consuming less than 3 meals per day, and those consuming less than 3 cups per day were designated as the reference group, respectively. The univariate analysis revealed that individuals consuming 3 or more meals per day showed a lower likelihood of GERD symptoms, with an odds ratio of 0.7, whereas those consuming 3 or more cups per day demonstrated a higher likelihood of GERD symptoms, with an odds ratio of 2.01. However, these associations did not reach statistical significance in the multivariate regression analysis after adjusting for other variables.

## Discussion

This first study in Egypt that aimed to investigate the prevalence of gastroesophageal reflux disease (GERD) among Egyptian medical students and to link GERD to certain important determinants, such as lifestyle, and diverse risk factors in order to point out the prominent risk factors that need further attention.

According to this study, it was found that 17.1% of medical students in Egypt had GERD, which is regarded as relatively high. This is consistent with a comprehensive review that found GERD affects between 8.7 and 33.1% of the Middle Eastern population [19]. This percentage is lower than the 33.2% reported among university students in Saudi Arabia’s southwest [20]. Another study at Shaqra University in Saudi Arabia found a prevalence of 23.8% [21]. Both studies used GerdQ questionnaire similar to our study to ascertain the diagnosis of GERD. The prevalence of GERD varies greatly over the world, ranging from 2.5 to 7.8% in East Asia, 8.7-33.1% in the Middle East, 8.8-25.9% in Europe, 18.1-27.8% in North America, 23.0% in South America, and 11.6% in Australia [19].

The prevalence of GERD, in the current study, is lower than those of the majority of prior Arab-region studies [20–22]. This could be attributed to the nature of our participants’ education, which may have influenced their awareness and attitude regarding GERD symptoms and potential risk factors [23]. Another possibility, considering the current study, is the lower prevalence of current smokers.

Age was not a significant determinant of GERD in our study. This finding is in agreement with a previous study conducted in Saudi Arabia, in which they found no correlation between age and GERD [21]. But contradicts another study conducted also in Saudi Arabia [24]. This may be due to the younger age group of our study participants. Additionally, there is no significant association between sex and GERD. This contradicts earlier studies suggesting females are more likely than males to have GERD [1, 9] as well as a study that found GERD is more prevalent in males [20].

Concerning the increased risk of GERD in smokers, a prior study reported a significant association between GERD and current tobacco smokers [24]. Furthermore, previous studies have revealed the benefits of smoking cessation in the treatment of GERD symptoms [25].

In the current study, 23.7% of medical students experienced high levels of perceived stress, and participants who experienced high levels of perceived stress were more likely to develop GERD.

Psychological stress is a significant risk factor that has been shown to aggravate GERD symptoms through a variety of methods [8]. Previous studies have found a link between stress and reflux esophagitis [7, 8, 26]. It is said to be more prevalent in medical students due to the constant burden of study and assessments [11]. Stress causes gastric acid output to increase, gastric emptying to slow and delay, and reflux to occur [8]. Furthermore, a prior study found that most GERD patients experienced increased symptoms when confronted with stressful circumstances [7].

It is important to note that our study utilized the GerdQ questionnaire, which cannot differentiate between GERD and functional heartburn. Therefore, based on these findings, we can only conclude that there is an association between stress and subjective symptoms of GERD, regardless of the presence of true acid reflux.

This study revealed that GERD family history is significantly associated with GERD and is an independent predictor of it. This finding is consistent with other studies in Saudi Arabia [21, 22] and may point to genetic factors or exposure to the same risk factors.

Our study showed that dietary habits such as food type, eating dinner one hour or two hours before sleeping, and eating meals at midnight are not significantly associated with GERD. In contrast to another study in Sri Lanka, poor food habits were observed to be associated with GERD symptoms [27]. But this is consistent with a study conducted at Shaqra University in Saudi Arabia, in which the authors stated that diets high in fatty and spicy foods did not demonstrate a significant association with GERD [21].

Obesity had no significant association with GERD in our study. This contradicts the findings of an Indian prospective cross-sectional study, which found that the prevalence, frequency, and severity of GERD symptoms increase with increasing Body Mass Index (BMI) [28]. The lack of a significant association between obesity and GERD in our study could be attributed to several factors. These include the reliance on self-reported weight measurements, potential lack of representativeness of the sample compared to the general population, and the possibility that the weight variations among college students might not accurately reflect the broader population’s variations. Additionally, chance variation could also contribute to the observed lack of association.

In terms of medications, Proton Pump Inhibitors (PPI) demonstrated a significant association with GERD. A greater proportion of students with GERD used PPIs to alleviate symptoms. This observation is consistent with the findings of another Indian study [9].

## Limitations

The study had certain drawbacks. Because of the cross-sectional design, it is impossible to ascertain the direction of the connection between the risk factors studied and GERD. Therefore, we recommend conducting larger prospective studies to address and investigate the modifiable factors related to the condition. Data were collected through self-report questionnaires. Recall bias may have influenced the results.

Another significant drawback of the study is its reliance on the GerdQ questionnaire, which depends on self-reported symptoms and lacks objective assessment. Nevertheless, despite this limitation the GerdQ questionnaire demonstrates comparable levels of sensitivity (65%) and specificity (71%) to the diagnoses made by gastroenterologists. In comparison, gastroenterologists reported a sensitivity of 67% and specificity of 70% [29].

## Conclusions

GERD is a common problem among medical students in Egypt, affecting approximately one-fifth of medical students. Because of the widespread prevalence of GERD, its consequences on everyday life, the young age of the individual investigated, and the potential for future problems, this illness may pose a significant health and economic burden. Stress, smoking, and family history were independent predictors of GERD. As a result, introducing smoking and stress management programs at colleges may be effective in the fight against GERD. Moreover, there should be public health campaigns to spread information about the disease and its risk factors.

## Data Availability

The data of this study are available from the corresponding author upon reasonable request.
